# Langya virus: Slope of the iceberg for unexplored pathogens

**DOI:** 10.1097/JS9.0000000000000151

**Published:** 2023-01-23

**Authors:** Ruhul Amin, Ronald Darwin, Hitesh Chopra, Talha B. Emran

**Affiliations:** a Faculty of Pharmaceutical Science, Assam Down Town University, Panikhaiti, Guwahati, Assam, India; b Department of Pharmacology, School of Pharmaceutical Sciences, Vels Institute of Science Technology & Advanced Studies, Chennai, India; c Chitkara College of Pharmacy, Chitkara University, Punjab, India; d Department of Pharmacy, BGC Trust University Bangladesh, Chittagong, Bangladesh; e Department of Pharmacy, Faculty of Allied Health Sciences, Daffodil International University, Dhaka, Bangladesh

Highlights

- Langya virus is a *Henipavirus* that has been found in shrews, bats, and rats.
- The Langya virus’s origin is still unknown.
- Henipaviruses do not have any approved therapies.
- There is currently no evidence that the virus may spread from human to human.

*Dear Editor*,

Langya virus is a *Henipavirus* that has been found in shrews, bats, rats, and other small animals. Some studies also reveal that animals such as dogs and goats have natural antibodies to the Langya virus^1^. Furthermore, when exposed to bat urine, the virus may spread to other species such as horses or infect pigs^2^. As a member of the RNA virus family *Paramyxoviridae*, the Langya henipavirus (LayV) has evolutionary ties with Nipah virus and Hendra virus^3^. Both the Nipah and Hendra viruses are emerging zoonotic diseases in the Asia-Pacific area. Despite their differences, these two RNA viruses have antigenic, serological, and ultrastructural features that place them in the same genus^4^.

On 4 August, it was initially reported in *The New England Journal of Medicine*. Thirty-five persons in China’s Shandong and Henan provinces contracted the LayV between December 2018 and May 2021^5^. This indicates that the virus is not spreading among humans readily. There have also been no clusters of instances within the same family, like what may happen with coronavirus disease 2019, or clusters within a short amount of time or in close proximity to one another^4^,^6^.

Contact tracing was performed on nine of the affected persons, and after contacting 15 close contacts of each patient, the researchers found no evidence of LayV infection^7^. This finding lends credence to the theory that the virus is transmitted not from person to person but from animal to human. To spread the Langya virus from person to person still needs a great deal of effort and direct physical contact. People with fever in eastern China who had just been exposed to animals led to the discovery of the virus. In the absence of a cure, problems may be treated with supportive care.

Scientists investigated different animals for the presence of the Langya virus to determine the origins of the infection. They discovered viral evidence in goats and dogs, but the shrew was the principal source of the Langya virus. The virus was found in 27% of the shrew samples^8^.

The Langya virus’s origin is still unknown. However, it is most likely passed from animal to human. Because the LayV virus RNA has been identified mostly in shrews, they may be its natural hosts^9^. The virus’s contagiousness is determined by its spreadability and contagiousness. There are currently no facts about the human-to-human transfer. The scholars assert that the investigation’s sample size is too small to detect any transmission. Conversely, no close-contact LayV transmission was found after tracking the contacts of nine patients with 15 close relatives^2^.

Those infected have reported the following symptoms: fever, fatigue, cough, anorexia, myalgia, nausea, headache, and vomiting. Furthermore, 35% of those contaminated had impaired liver function, and 8% of those infected had impaired renal function. According to doctors, the virus might cause a decrease in the number of platelets in the blood, significant damage to the kidneys and liver, and possibly death^5^. The Langya virus may produce symptoms such as fever, cough, fatigue, appetite decrease, muscle cramps, headache, and vomiting.

Serum seropositivity was found in 2% of goats and 5% of dogs in a sample of domestic animals^10^. Since the identification of this Henipavirus strain is novel, it serves to illustrate the ever-present danger of the creation of (new) infections. Due to the small sample size, further work is needed to fully grasp the disease’s epidemiological and microbiological features. When it comes to combating potential pandemic infections, time is of the essence, making surveillance for their development a crucial tool.

Henipaviruses do not have any approved therapies. In animal experiments, experts have only tested a few antiviral alternatives. There is also no particular vaccination for the Langya virus. However, ribavirin may be an effective therapy. Doctors often use this medicine to treat viral infections that have no other therapeutic options. Ribavirin is effective against RNA viruses, including those that cause respiratory problems. Studies demonstrate that ribavirin is effective for both Hendra and Nipah viruses. The malaria medicine chloroquine may also be useful in treating these two conditions^1^,^11^. As a result, if necessary, these two medications may aid in the control of the Langya virus.

This unusual virus is poorly understood, and experts predict that the number of verified cases will rise. There is currently no evidence that the virus may spread from human to human. More study is required to determine the virus’s entire scope, its mode of dissemination, and whether it has already reached China and the surrounding region.

## Ethical approval

Not applicable.

## Conflicts of interest disclosure

There are no conflicts of interest.

## Sources of funding

No funding was received.

## Author contribution

R.A.: conceptualization, data curation, writing – original draft preparation, reviewing, and editing. R.D. and H.C.: data curation, writing – original draft preparation, reviewing, and editing. T.B.E.: writing – reviewing and editing, visualization, and supervision.

## Research registration unique identifying number (UIN)

1. Name of the registry: not applicable.

2. Unique identifying number or registration ID: not applicable.

3. Hyperlink to your specific registration (must be publicly accessible and will be checked): not applicable.

## Guarantor

Talha Bin Emran, PhD, Associate Professor, Department of Pharmacy, BGC Trust University Bangladesh, Chittagong 4381, Bangladesh. Tel: +880 303 356 193, fax: +880 312 550 224. https://orcid.org/0000-0003-3188-2272

## Provenance and peer review

Not commissioned, internally peer-reviewed.

## Data statement

The data in this correspondence article is not sensitive in nature and is accessible in the public domain. The data is therefore available and not of a confidential nature.
